# Fragment-based screening with natural products for novel anti-parasitic disease drug discovery

**DOI:** 10.1080/17460441.2019.1653849

**Published:** 2019-09-12

**Authors:** Miaomiao Liu, Ronald J. Quinn

**Affiliations:** Griffith Institute for Drug Discovery, Griffith University, Brisbane, Australia

**Keywords:** 3D shape, anti-parasitic, chemical diversity, fragment-based drug discovery, malaria proteome, native mass spectrometry, natural products, natural product-like

## Abstract

**Introduction**: Fragment-based drug discovery can identify relatively simple compounds with low binding affinity due to fewer binding interactions with protein targets. FBDD reduces the library size and provides simpler starting points for subsequent chemical optimization of initial hits. A much greater proportion of chemical space can be sampled in fragment-based screening compared to larger molecules with typical molecular weights (MWs) of 250–500 g mol^−1^ used in high-throughput screening (HTS) libraries.

**Areas covered**: The authors cover the role of natural products in fragment-based drug discovery against parasitic disease targets. They review the approaches to develop fragment-based libraries either using natural products or natural product-like compounds. The authors present approaches to fragment-based drug discovery against parasitic diseases and compare these libraries with the 3D attributes of natural products.

**Expert opinion**: To effectively use the three-dimensional properties and the chemical diversity of natural products in fragment-based drug discovery against parasitic diseases, there needs to be a mind-shift. Library design, in the medicinal chemistry area, has acknowledged that escaping flat-land is very important to increase the chances of clinical success. Attempts to increase sp^3^ richness in fragment libraries are acknowledged. Sufficient low molecular weight natural products are known to create true natural product fragment libraries.

## 1. Introduction

In this opinion piece, we explore approaches to using natural products to build fragment libraries and identify examples of natural products that have been reported in other fragment-based libraries. We then discuss the parasitic disease targets that have been subject to fragment-based screening.

There has been a realization that 3D shape and sp^3^ richness may be better starting points for drugs [1] and, likewise, in fragment-based drug discovery efforts are being made to include these features into Fragment Screening Libraries [2–7]. Methods of chemical synthesis of fragments hits based on natural product-like compounds follow well-established principles of fragment-linking and fragment-growing. Tractability is enhanced by the small size and reduced complexity of fragment hits based on natural product-like compounds compared to synthetic challenges associated with larger natural products.

While the construction of libraries has been historically uneven in its exploration of chemical space [8], there has been only limited use of the incorporation of natural products into Fragment Libraries. Natural products are enriched with biosynthetic intermediates and endogenous metabolites resulting from being exposed to long selection processes. Natural products encode areas of chemical space explored by nature in evolution [4]. Natural products have an embedded recognition of protein binding sites captured during their biosynthesis [9–11].

A fragment’s effect on proteins is often too weak to be measured (affinity in the 0.1–10 mM range) using standard assays. The screening stage of FBDD requires the use of biophysical techniques significantly more sensitive than the methods utilized in HTS. There are sensitive techniques that are used in the FBDD such as X-ray crystallography, nuclear magnetic resonance (NMR), native mass spectrometry (NMS), computational approaches, thermal shift assay, and computational approaches.

A recent bibliographic technique identified 3,642 publications on FBDD between 1953 and 2016 [12]. The bibliographic analysis was based on keywords. Natural products showed up in the top 100 keywords; however, anti-parasitic did not appear, with the major disease-related keywords including cancer, apoptosis, kinase inhibitor, alzheimer’s diseases beta-secretase and gpcr [12]. The various techniques in fragment-based discovery were, in highest to lowest order, X-ray crystallography, surface plasmon resonance, nuclear magnetic resonance, thermal shift assay, isothermal titration calorimetry, mass spectrometry [12]. It was noted that keywords may not perfectly reflect the usage of the techniques as keywords tend to reflect identifiers used by authors to attract the targeted audience [12].

## 2. Approaches to develop fragment-based libraries either using natural products or natural product-like compounds

### 2.1. Chemical disassembly of larger natural products

**Figure 1.**
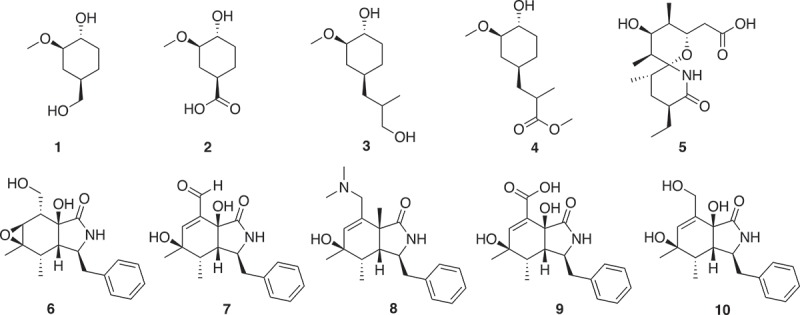
Fragments from FK 506 (Tacrolima) (**1–4**), sanglifehrin A (**5**) and cyctochalasin E (**6–10**).

*In silico* guided chemical disassembly of larger natural products has been developed as an approach to a natural product library [13]. Fragmentation using *in silico* cleavage reactions gave 66,000 virtual products from a starting library of 17,000 natural products. ‘Fragment-like’ criteria of 150 < MW < 300 and clogP < 3 reduced the number to 9,000. 3D shape assessment and novelty assessed using Extended Connectivity Fingerprints with a radius of 6 (ECFP6) gave the final set [14]. Fragments (**1**–**10**) from FK 506 (Tacrolima), sanglifehrin A and cyctochalasin E are shown in Figure 1. These fragments can be classified as 3D-shaped, natural product like fragments.

### 2.2. Chemical modification of smaller natural products

**Figure 2.**
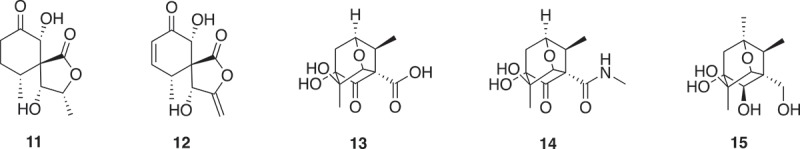
Compounds (**11–15**) obtained by derivatization of massarigenin.

**Figure 3.**
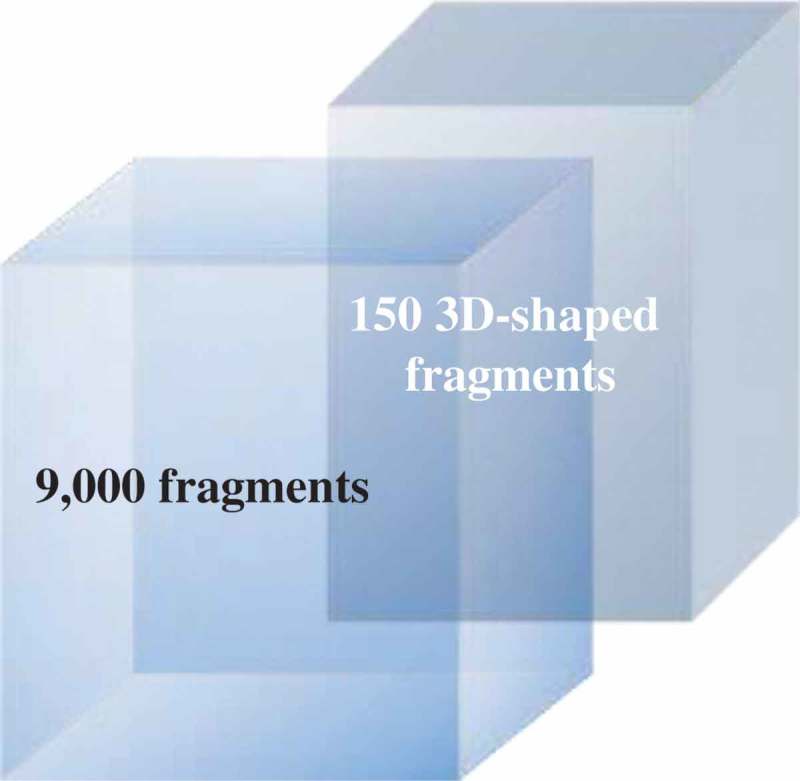
Shape (fraction of Csp^3^) and natural product likeness analysis of the generated fragment library (150 members) and current Novartis in-house fragment library (9,000 members) [13]. There is only minor overlap, the new small fragment library occupies unique chemical space. Adapted from reference [13].

In addition to deconstruction of high molecular weight natural products, chemical modification of smaller natural products with an objective to remove potential reactive sites and introduce novel chiral sp^3^ centers has been used (Figure 2) [13]. An analysis of natural product-likeness indicated that the two libraries occupied unique areas of chemical diversity compared to an already established Fragment library (Figure 3).

### 2.3. Design of “pseudo natural products”

**Figure 4.**
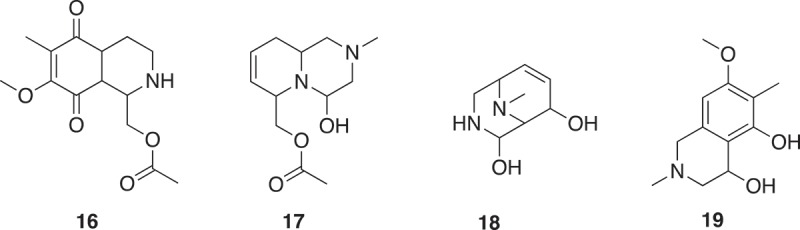
Two ring fragments from a Scaffold Tree analysis of renieramycin [4].

**Figure 5.**
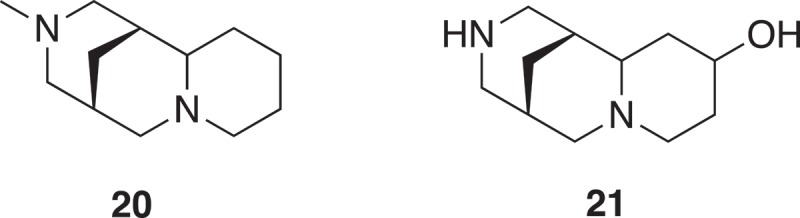
A fragment (**20**) of a cluster of 85 members and is a substructure of 53 natural products such as sparteine, fragment (**21**) is the cluster center of the 85 member cluster and is a substructure of bapitifoline.

With the objective to identify compounds which resemble natural products and are rich in sp^3^ centers, an approach has been to deconstruct natural products into fragments [4,15]. The logic and algorithm used was a scaffold tree approach, whereby identification of the scaffold was undertaken by removal of terminal chains and ring substituents followed by deconstruction, by removing rings and bonds successively following a set of prioritization rules ensuring linear deconstruction giving one fragment at each step. Thus, directly attached functional groups are kept intact, side chains are shortened, and ring systems are deconstructed in a stepwise manner. Side chains with lengths of up to two atoms are retained, and carbon chains longer than two atoms are pruned after the second atom from the ring. Where the second atom is a heteroatom, pruning will occur after the first following carbon. Carbonyl groups are treated as a single heteroatom. For rings separated by a linker, the connection is pruned and each substructure is then processed separately. The position and type of substitution are therefore retained as in the guiding natural product, and functional groups are differentiated. As fragments may not have a high Fsp^3^ or 3D shape, an NP-likeness score was subsequently calculated [16] to give 2,000 clusters of natural product derived fragments with structural diversity. The generation of fragments (**16**–**19**) from renieramycin is shown in Figure 4 [4,15]. The fragment (**20**) from a cluster, where **21** was the cluster center, was identified as weak inhibitors of p38α MAP kinase with an IC_50_ of 1.3 mM. Synthetic elaboration and subsequent co-crystal structures with the protein revealed an allosteric pocket of p38α MAP kinase as novel class of type III inhibitors (Figure 5) [15].

A scaffold network consists of all possible combinations that can be generated by pruning rings from the parent scaffold and covers scaffolds that are not covered in the scaffold tree approach [17].

**Figure 6.**
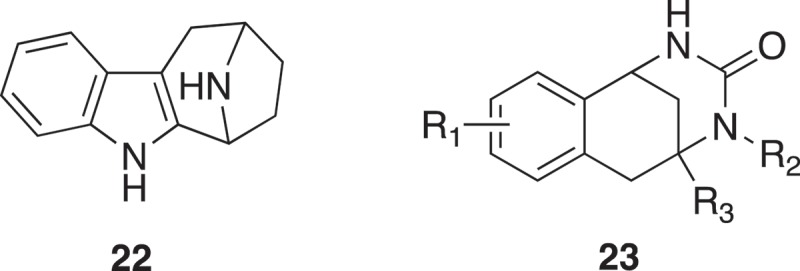
Indotropanes and chromopyrone ‘pseudo natural products’.

Synthetic combination of unrelated natural product fragment types is a new approach to the design of “pseudo natural products” (Figure 6). Two biosynthetically unrelated natural product fragment types are combined. Each scaffold structure encodes areas of chemical space explored by nature in evolution. De novo combination of NP fragments will go beyond the areas of chemical space explored by nature and can be regarded as “pseudo natural products”. Based on the previous study [4], synthetic programs yielded collections of NP-inspired compounds. Analysis of chemical space indicated that indotropanes occupy an area of natural product space not accessible via known biosynthetic pathways [18]. The “indotropanes” (**22**) were produced by combination of indole and tropane scaffolds (Figure 6). The screening approach was via cell-based assays (target-agnostic) followed by target identification to identify myokinasib as the first selective, isoform-specific inhibitor of myosin light chain kinase 1 (MLCK1) inhibitor [18].

A further proof of principle was obtained by combining the biosynthetically unrelated chromane and tetrahydropyrimidinone NP fragments. This was modeled on the reassembly of biosynthetic pathways into new artificial biosynthetic pathways [19]. Biological investigation of a synthesized compound collection revealed that the chromopynones (**23**) (Figure 6) define a truly novel, structurally unprecedented glucose uptake inhibitor chemotype that selectively targets glucose transporters GLUT-1 and GLUT-3 for modulation of tumor metabolism [20].

**Figure 7.**
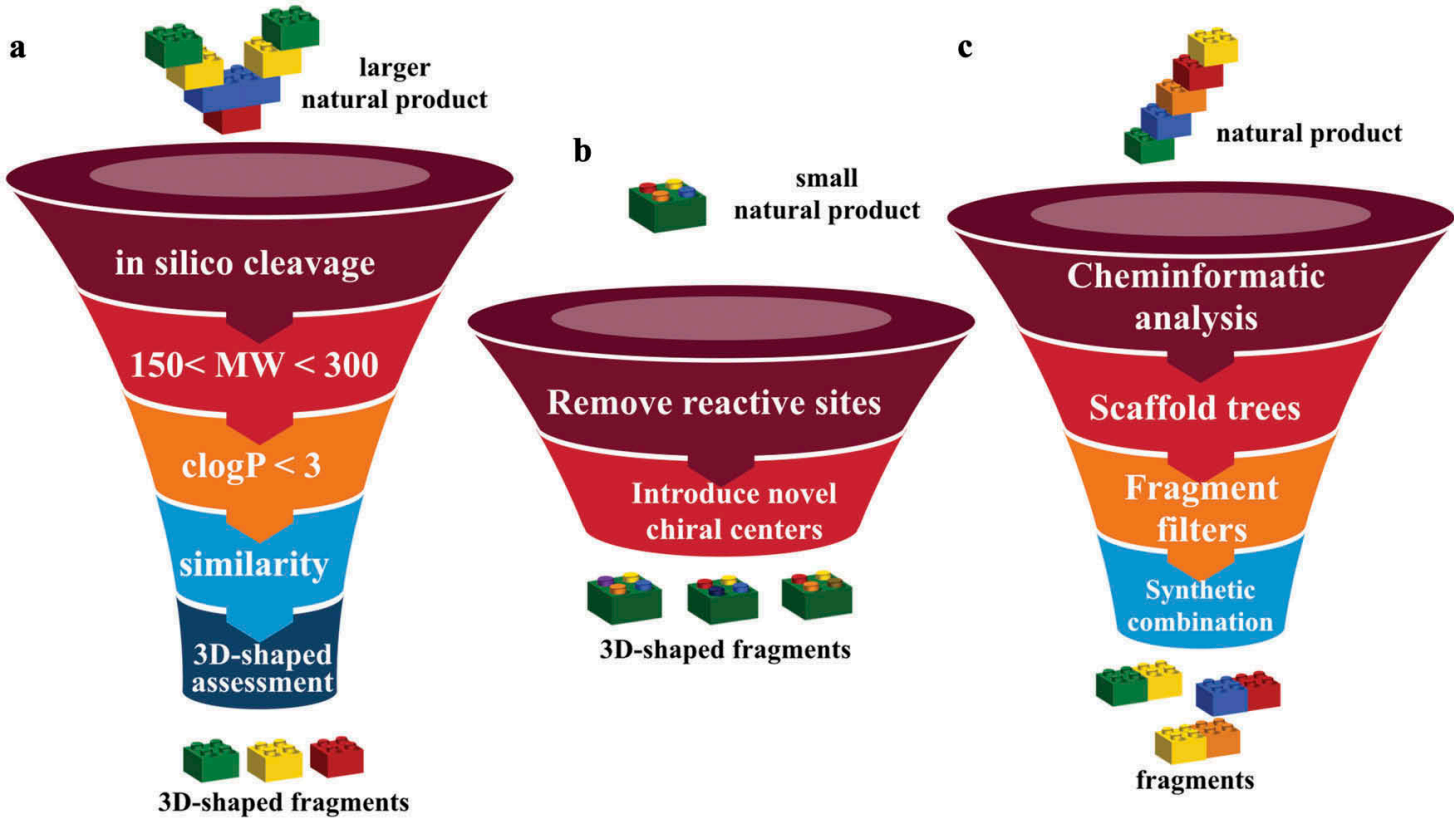
(a) *In silico* guided construction of 3D-shaped natural product fragment library. (b) construction of 3D-shaped natural product fragment library by chemical modification of small natural products. (c) Design of “pseudo natural products” fragment library.

These approaches of chemical disassembly of larger natural products, chemical modification of smaller natural products and design of “pseudo natural products” are illustrated in Figure 7.

### 2.4. Virtual design of library

Target prediction of fragment-like natural products with innovative scaffolds may be used as starting points for chemical biology and medicinal chemistry programmes, as recently exemplified through the development of natural-product-inspired synthetically accessible anticancer compounds [21,22]. Software has been developed to handle natural-product-derived scaffolds and identify areas in natural product chemical space that are correlated with certain bioactivity profiles [23,24]. A key feature of these tools is the visualization of distributions of natural products and synthetic bioactive compounds (sometimes referred to as ‘chemography’), which facilitates the identification of promising molecular scaffolds for further exploration [25,26].

The Dictionary of Natural Products database has been computationally divided into 64,650 fragment sized (MW 100–300 Da) and 145,623 natural products with an MW > 300. SPiDER software [27] predicted the targets of 23,340 (36%) of the low MW natural products compared to 31,556 (22% of the 145,623 larger natural products). The concept of target prediction for fragment-like natural products through a comparison with drug-like small molecules was undertaken with sparteine for a prospective experiment. In addition to weakly inhibiting p38α mitogen-activated protein kinase, sparteine was previously shown to bind to the muscarinic and nicotinic receptors [28]. SPiDER predicted these two targets among the top three predictions. The kappa opioid receptor was listed as the second-most-confidently predicted target. Binding and functional assays confirmed the prediction and revealed sparteine as a ligand-efficient fragment for further development (ligand efficiency = 0.30) [29].

### 2.5. Design of low MW library

**Figure 8.**
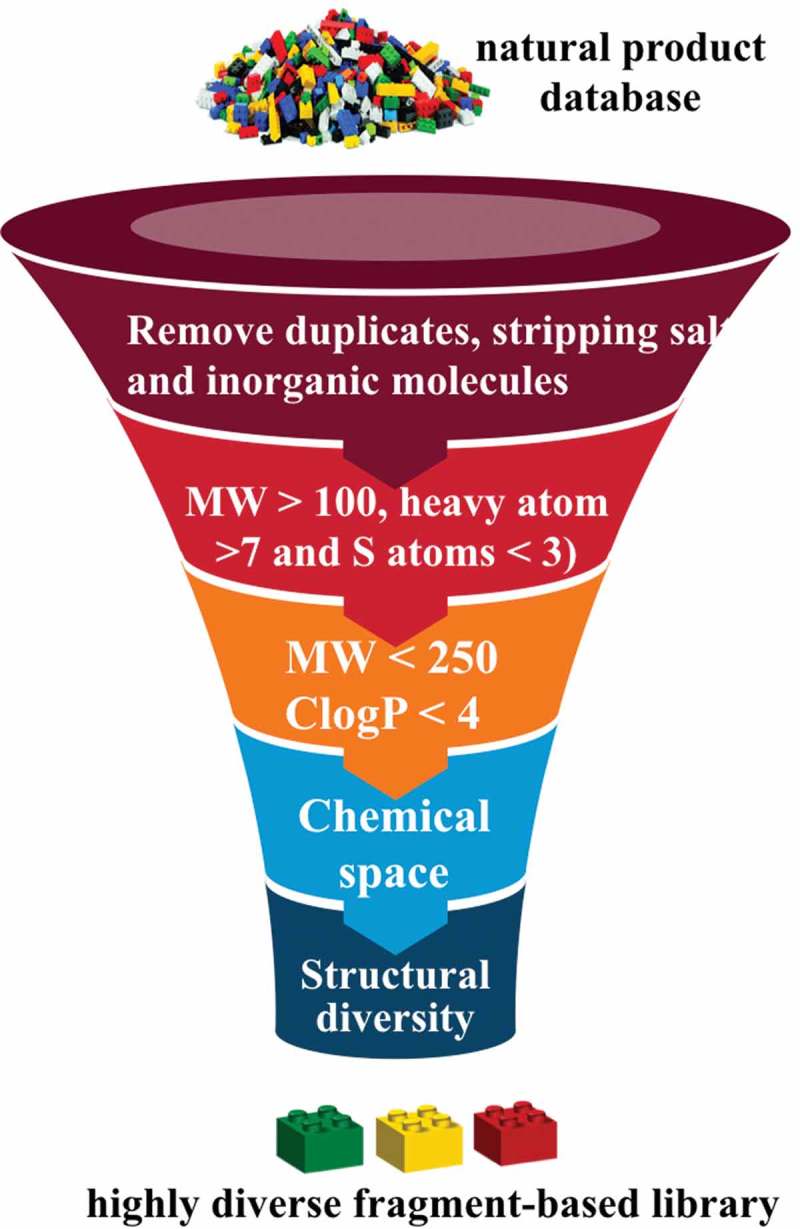
Workflow of a low MW natural product library design.

**Figure 9.**
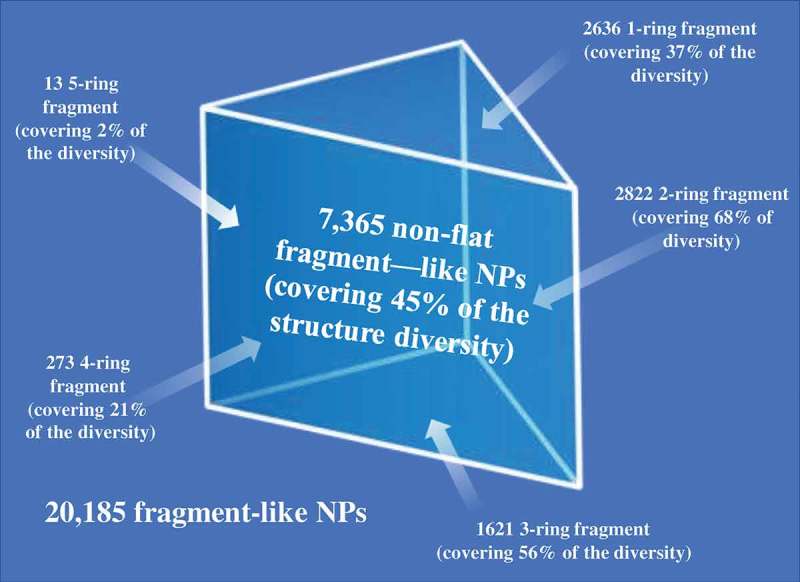
Overlay of 7,365 non-flat fragment-sized natural products on a SOM trained using ECFP_4 of 20,185 fragment-like natural products and the coverage of non-flat fragments with different ring systems in terms of structure diversity.

**Figure 10.**
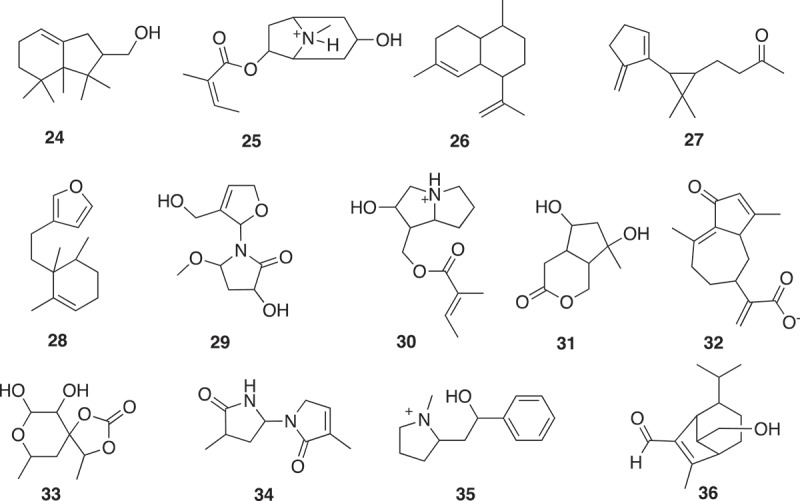
Representative seed compounds of cells from the SOM analysis in Figure 9.

The foundation for establishing a fragment screening library of low molecular weight natural products originated from a Dictionary of Natural Products analysis that found 7,365 non-flat fragment-sized natural products rich in sp^3^ centers (figures 8 and 9) [5]. It was shown that fragment-sized natural products cover ~66% of the pharmacological features found in natural products [5]. The identification of non-flat fragments from 20,185 fragments was carried out through calculation of the complexity of molecules in terms of carbon bond saturation (Fsp^3^*). A set of 7,345 non-flat fragments were identified with Fsp^3^* >0.45. Using a self-organizing map to compare structure diversity between both datasets indicated 45% of the whole fragment structure diversity was covered by the non-flat fragments. Additionally, the analysis identified 2,822 two-ring natural products covering 68% of them non-flat fragment diversity using a self-organizing map calculation. The study highlighted the potential of natural products with fragment-like physicochemical parameters to capture the structural diversity of nature. Several examples of the non-flat-2-ring natural product fragments are shown in Figure 10.

**Figure 11.**
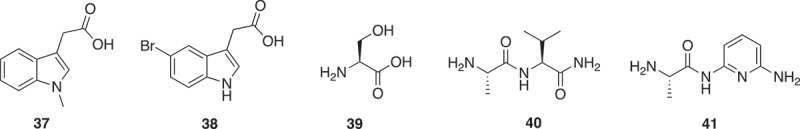
Examples of natural products that have provided hits.

**Figure 12.**
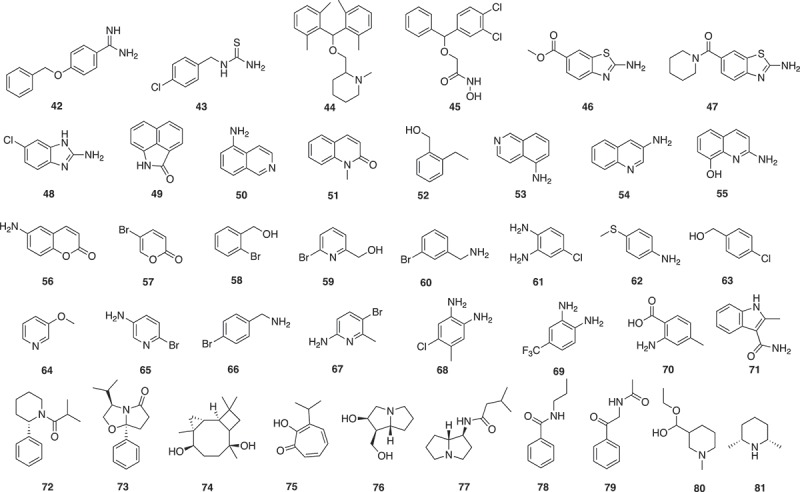
Fragment-based hits.

**Figure 13.**
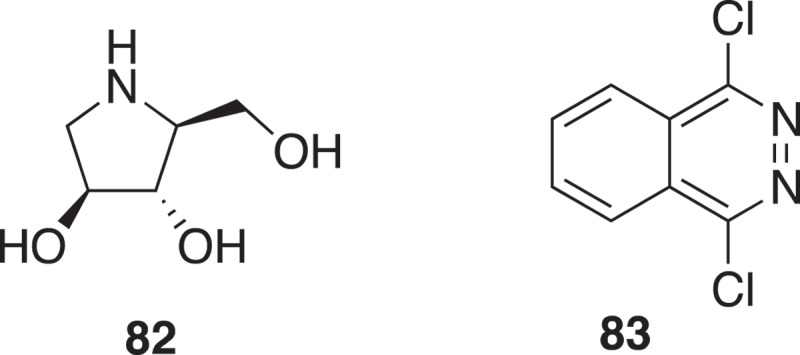
Fragments from a 1-deoxynojirimycin-based library and a Ro3 library.

A library of 643 natural products with fragment-like physicochemical properties was assembled as a screening library [30].

### 2.6. Natural products in other fragment libraries

Other Fragment Libraries, while not designed on natural products, may include a low proportion of natural products. Fragment screening using X-ray crystallography can identify secondary sites that may have a biological function. Two analogs (**37, 38**) (Figure 11) of indole-acetic acid, which is a plant hormone, bound within the nucleic acid binding groove of viral protease-helicase protein HCV NS3. This work implies that the opportunities for modulating protein function with small molecules via such sites are far more widespread than previously assumed. Many secondary sites remain unknown and therefore offer potential for novel approaches to modulate these protein targets [31]. A fragment-based crystallographic screen versus the M2 isoform of human pyruvate kinase (PKM2) revealed the amino acids L-alanine, L-cysteine, L-threonine and L-serine bound to a previously uncharacterized binding pocket on PKM2. Crystallographic soaking experiments revealed L-serine (**39**) (Figure 11) bound to PKM2, with a single L-serine molecule bound to each of the monomers comprising the PKM2 tetramer [32]. This led to elucidation of the mechanism that tightly controls the metabolic bifurcation of glucose-derived carbon. When serine is abundant, PKM2 is fully active, enabling the maximal use of glucose through glycolysis. When serine levels are low, PKM2 activity is attenuated. This enables the fast shuttling of glucose-derived carbon to serine biosynthesis, compensating for the serine shortfall and enabling growth and proliferation in the absence of these amino acids. Finally, by activating PKM2, serine supports aerobic glycolysis and lactate production, events that are critical for cancer cell growth and survival [33].

Inhibitor of apoptosis proteins (IAPs) are important regulators of apoptosis and pro-survival signaling pathways whose deregulation in various cancers is associated with tumor growth. A fragment-based screening approach, using ^1^H NMR spectrum to detect fragment binding by monitoring the chemical shifts and line widths of XIAP-BIR3 ^1^H signals with δ < 0.4 ppm, where none of the fragments had 1H NMR signals. In addition, the range δ 9.8 − 10.4 ppm was also monitored; where XIAP-BIR3, has four signals due to the indole NH protons of tryptophan side chains. One thousand one hundred and fifty-one fragments were screened in pools of two, with each fragment at 10 mM. Hits were followed-up by a ^1^H−^15^N HSQC NMR experiment using ^15^N labeled XIAP-BIR3. Hits were soaked into XIAP crystals for X-ray crystallography. The alanine based fragments (**40**) and (**41**) (Figure 11) had the best ligand efficiency. The binding mode of **40** and **41** was used for hypothesis-driven optimization of a nonalanine hit, resulting in potent nonalanine IAP antagonists structurally distinct from all IAP antagonists previously reported. The lead compound had activity in cell-based assays and in a mouse xenograft efficacy model and represents a highly promising start point for further optimization [34].

## 3. Approaches to fragment-based drug discovery against parasitic diseases

### 3.1. Multiple parasitic species

Five hundred (500) drugs tested in the literature against some species out of a list of 16 parasite species and 207 drugs that were not tested were analyzed using multi-target spectral moment QSAR in combination with a fragment-based topological approach. The data were processed by linear discriminant analysis (LDA). The model correctly classified 311 out of 358 active compounds (86.9%) and 2328 out of 2577 non-active compounds (90.3%) [35]. The 30 fragments were predominantly flat lacking the sp^3^ richness of natural products.

Perturbation theory combined with machine learning (PTLM) models is advanced versions of the well-known QSAR (quantitative structure–activity relationships) models. A distinctive aspect of the PTML models is that they enable the calculation of the quantitative contribution of any molecular fragment to the activity, toxicity, and/or ADME property under analysis. PTML models have been successfully applied to fragment-based drug discovery within different therapeutic areas. The multitarget modeling methodology focused on the joint use of an ML model with a fragment-based topological design approach might serve as a powerful alternative to speed up early drug discovery [36].

The area of fragment-based drug discovery as an alternate strategy for drug development for neglected disease has been reviewed in 2017 [37]. We have analyzed this review and more recent publications to identify fragment Hits against parasitic diseases.

### 3.2. T. brucei, T. cruzi

A combined fragment-based approach and a phenotypic approach have been undertaken for antiparasitic activity [38]. An in-house library of 1,040 fragments was screened by a bioluminescence phenotypic assay on *Tbr*PDEB1, which is an essential cyclic nucleotide phosphodiesterase (PDE) from *Trypanosoma brucei*. Twelve fragments were found to display more than 90% inhibition activity against *Tbr*PDEB1 and seven of them showing selective for *Tbr*PDEB1 inhibition over human PDE4D. Four fragment hits (**42**–**45**) (Figure 12) with low micromolar range against *Tbr*PDEB1 (4.0–4.9 μM) were selected for further analog development and evaluation due to their high similarities with known active drug scaffolds. The analogs were screened against a phenotypic panel of *T. brucei, Trypanosoma cruzi, Leishmania infantum*, and *Plasmodium falciparum*, as well as MRC-5 human lung cells and two analogs displayed slightly higher antiparasitic activity and low cytotoxicity on human MRC-5 cells.

Fragment-based drug discovery using virtual screening targeted *T. brucei* pteridine reductase 1 (*Tb*PTR1), a potential target to treat human African trypanosomiasis, enzyme [39]. A serial of physico-chemical property filters, including less than 20 heavy atoms, less than two ring systems, at least one hydrogen-bond donor, less than four rotatable bonds, and a ClogP/ClogD ratil below 3.5 was applied to a commercially available library of 250,000 compounds, resulting in 26,084 fragment-like molecules. The fragment library was docked into the *Tb*PTR1 binding site and ranked based on docking scores. Subsequent filters, such as dissimilarity with known inhibitors and PSA less than 70 A, were used to generate 59 compounds for experiment test. Three fragments (**46**–**48**) (Figure 12) showed more than 50% inhibition of *Tb*PTR1 at concentration of 100 μM and two were available for further analog development. The most potent phenyl-derivative was more than 1,500-fold more active than the original fragment hit (**48**) with an apparent K_i_ (K_i_^app^) of 7 nM and highly selective over both human and *T. brucei* DHFR.

Crystallographic fragment screening is one of the major techniques for FBDD in which protein crystals are soaked with high concentrations of fragments. Specific binding of ligands to the protein is detected from analysis of X-ray diffraction data collected from crystals. The main advantage of X-ray crystallography is it can provide an immediate model of the fragment binding to the protein, however, the main disadvantage is that it requires a suitable crystal system for compounds bound to target proteins that can be a major issue for some targets [40]. Crystallographic fragment screening was used to find inhibitors of *Trypanosoma brucei* nucleoside 2-deoxyribosyltransferase (*Tb*NDRT) [41]. Thirty-one fragment cocktails were generated from 304 commercially available fragments by mixing 7–10 compounds into one cocktail based on chemical properties. Co-crystallographic screening of the 31 cocktails with *Tb*NDRT resulted in 69 crystals, in which four ligands (**49**–**52**) (Figure 12) were identified in the active site. The four ligands were screened against bloodstream form *T. brucei* cell cultures, all showing growth inhibition effects with ED_50_ between 0.12 and 1.34 mM.

A similar study using crystallographic fragment screening was undertaken to find potential inhibitors of *T. cruzi* histidyltRNA synthetase (HisRS) [42]. Sixty-eight fragment cocktails were generated from 680 commercially available fragments by mixing 10 compounds into one cocktail. Crystallographic fragment screening of 68 cocktails with *Tc*HisRS*His crystals has identified 15 cocktails containing putative hits. Subsequent confirmation screening was performed by single soaking of all of the candidate fragments and 15 single fragments (**53**–**67**) (Figure 12) were confirmed as binders. All 15 fragments were found to bind to the same binding site of *Tc*HisRS*His, with one of the fragments (**55**) occupying a second binding site with lower affinity. Thermal shift assay was applied to all 15 hits, but only one fragment (**54**) showed thermal stabilization effect of *Tc*HisRS*His by about 1.6 ℃. Three fragment binders (**54, 55, 59**) were found to have weak inhibition activity (20%, 21%, and 39%) in the aminoacylation assay at concentration of 2 mM.

Most NMR approaches adopted in fragment-based screening focus on physical binding and not on the biochemical assay. Factorial analysis has allowed two approaches to study the enzyme reaction. Real-time monitoring of substrate-product using NMR requires ~1 hour of successive NMR measurements for each concentration resulting in several hours to measure each inhibitor. The constant time batch reaction used a thermal cycle unit to denature the protein thereby stopping enzyme action. The method was used to study spermidine synthase of *Trypanosoma cruzi* [43].

At the screening stage, a higher concentration of substrate and fewer points for inhibitor concentration allowed a reduction to 5 min using 5–10 points. This, 0.5–1 h should be required to evaluate a single compound, and screening of a fragment library of 1,000 compounds will take 20–40 days. The time for the screening will be drastically reduced if mixtures of compounds are used. It would take 2–4 days for 100 mixtures containing 10 compounds in each pool [43].

The method has been refined to allow fragment-based screening of the *Trypanosoma cruzi* oxidosqualene cyclase. In this case, the method was validated using one known and three novel inhibitors. The factor analysis allows the detection of nonlabelled intact molecules and can deal with mixed samples. Assuming that approximately 10 min is required for NMR measurement of one compound, a fragment library of 1,000 compounds will take 1 week. Using mixtures of 10 compounds and factor analysis score vectors, the initial screening can be accomplished in 1 day. The fragment-based screening remains to be reported [44].

### 3.3. Dengue virus

Fragment-based screening has been used to find new inhibitors targeting dengue virus helicase (NS3 DENV Hel) and dengue virus methyltransferase (NS5 DENV MTase), both involved in viral replication [45–47]. Thermal shift assay was performed to screen 500 fragments acquired from the Maybridge RO3 fragment library against dengue virus helicase (NS3 DENV Hel) and dengue virus methyltransferase (NS5 DENV MTase). In the thermal shift assay, 36 fragments showed melting temperature increase with NS3 DENV Hel and 32 fragments with NS5 DENV MTase, related to the protein folding stabilization by ligand interactions. Only the MTase responded well to the downstream crystal soaking [45]. NS5 AdoMet-dependent mRNA methyltransferase (MTase) is a viral protein involved in flavivirus replication. Specifically, no antiviral drugs are available to treat the life-threatening flavivirus infections caused by Dengue or Zika viruses. The viral replication/transcription is performed by five nonstructural (NS) proteins. MTase is at the N-terminus and catalyzes two methylation reactions. Seven fragment hits were identified by a primary fragment-based screening using thermal-shift assay followed by a fragment-based X-ray crystallography screening. Three of the Hits (**68**–**70**) (Figure 12) were at allosteric sites [45]. Computational methods were used to design linkers for these allosteric binders based on X-ray structures leading to a novel series of non-nucleoside inhibitors from which the core structure of one of these was used for further fragment-growing optimization. This gave two inhibitors with low micromolar activity that almost reached a 10 μM threshold. The MTase activity with IC_50_ values ~20 mM (DENV IC_50_ 26 μM and 24 μM; ZIKV IC_50_ 28 μM and 19 μM, respectively). The results validate a suitable allosteric site for the development of new classes of inhibitors targeting flaviviral cap MTases [47].

A combination of homology modeling, fragment docking, chemical similarity, and structural filters was used to identify hits against a homology model of DENV NS2B-NS3 protease, which was generated based on five WNV and DENV protease template structures [48]. A Fragment library of 149,151 fragments was generated from 14 million compounds of the ZINC screening database by filtering with the Rule-of-Three. Parallel high-throughput docking was applied to the entire fragment library, with a particular interest of two pockets S1 and S2, two sets of high-scoring binding fragment, each containing 110 fragments, were selected based on the S1 and S2 binding scores. Key structure features of binding fragment of the S1 and S2 pocket were indole (or other bicyclic systems) and positively charged molecular fragments under neutral condition, respectively. The 220 high-scoring fragments were subsequently submitted to the ZINC database for a substructure search, which resulted in 352,758 hits with preferred either S1 or S2 pocket binding fragment features. A number of hit compounds were removed to avoid chemically infeasible molecules, such as spiro-compounds or compounds with more than 19 bonds or with more than 5 rings. In addition, all compounds were required to contain a terminal cationic or basic moiety for favorable interactions with the S2 pocket, resulting in 18,803 hits. The number of hits was further reduced to 554 by dissimilarity clustering and subsequent manual inspection to select molecules with both S1 and S2 pocket binding fragments features but at opposite ends. Substructure search and dissimilarity clustering processes were repeated and resulted in 261 additional compounds. The final collection of 815 hits were re-docked with the DENV protease model and 23 promising candidates were picked for *in vitro* protease inhibition assay based on visual inspection. Two compounds were found to inhibit both DENV2 and West Nile virus (WNV) protease, one with IC_50_ of 7.5 μM against DENV2 and 6.3 μM against WNV while the other compound with IC_50_ of 37.9 μM against DENV and 39.0 μM against WNV. Both compounds are linked from a preferred binding fragment (**71**) (Figure 12) of the S1 pocket with a indole amide moiety.

Molecular docking has been used in a fragment-based drug design study to identify natural product inhibitors of the β-OG pocket binder of Dengue virus (DENV) envelope protein. (reference: Identification of natural products as an inhibitor of β-OG pocket binder of dengue virus envelope protein using fragment-based drug design and molecular docking approach [49]. A total of 190,084 natural product compounds were obtained from ZIN15 database. To obtain a library of fragment-like natural products, this dataset was further filtered with Ro3 (MW < 300, cLogP < 3, H donors <3, H acceptors <3), the Veber rules (rtB <3, TPSA < 60 Å) and the toxicity prediction parameters (Druglikeness higher than 0, no mutagenic, no tumorigenic, no reproductive effective, and no irritant). By applying these filters, the initial set was reduced to 1,610 low MW natural products. The resulting fragment library was sequentially docked into the β-OG pocket of DENV) envelope protein. Ten fragment binders (**72**–**81**) (Figure 12) with atoms in the middle cavity of the β-OG pocket and faced the other fragment from the opposed region were selected for future evaluation.

Fragment-based drug design for host endoplasmic reticulum α-glucosidase II inhibitors for dengue fever treatment used two fragment libraries. One fragment library was constructed from natural products with a Tanimoto similarity of 0.6 to 1-deoxynojirimycin to give **82** (Figure 13). The other Ro3 library gave **83** (Figure 13). The fragments were linked *in silico* [50].

### 3.4. P. falciparum

**Figure 14.**
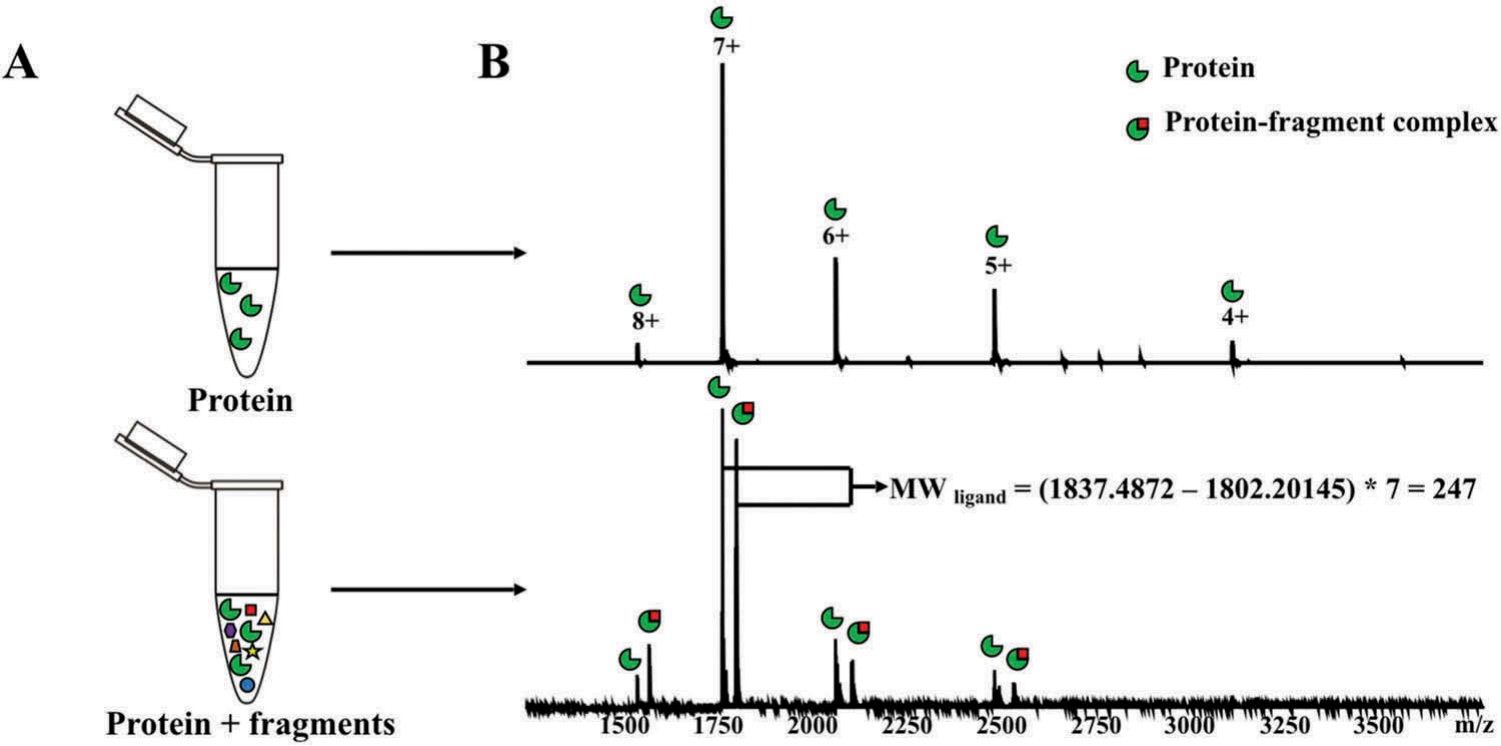
General workflow of a native mass spectrometry experiment for fragment screening. A. The target protein is incubated with a mixture of fragments. The negative control experiment is performed with protein only. B. Native mass spectrometry spectra acquired from both experiments. Protein-fragment complexes are detected by comparing with the control spectrum.

Native mass spectrometry provides key advantages for weak binding detection as it has high sensitivity, low sample consumption, does not require modifications or labeling of the protein target, and provides a direct visualization of all species present in solution under binding equilibrium [51,52]. The technique relies on non-denaturing electrospray-ionization (ESI) to recognize multi-charged proteins in their near-native states. High resolution, high mass accuracy measurements, coupled with soft ionization techniques to preserve the integrity of complexes, allows for the determination of ligand mass by measuring the mass of the protein and the mass of the intact protein:ligand complex. The general workflow of a native mass spectrometry experiment for fragment screening is shown in Figure 14.

**Figure 15.**
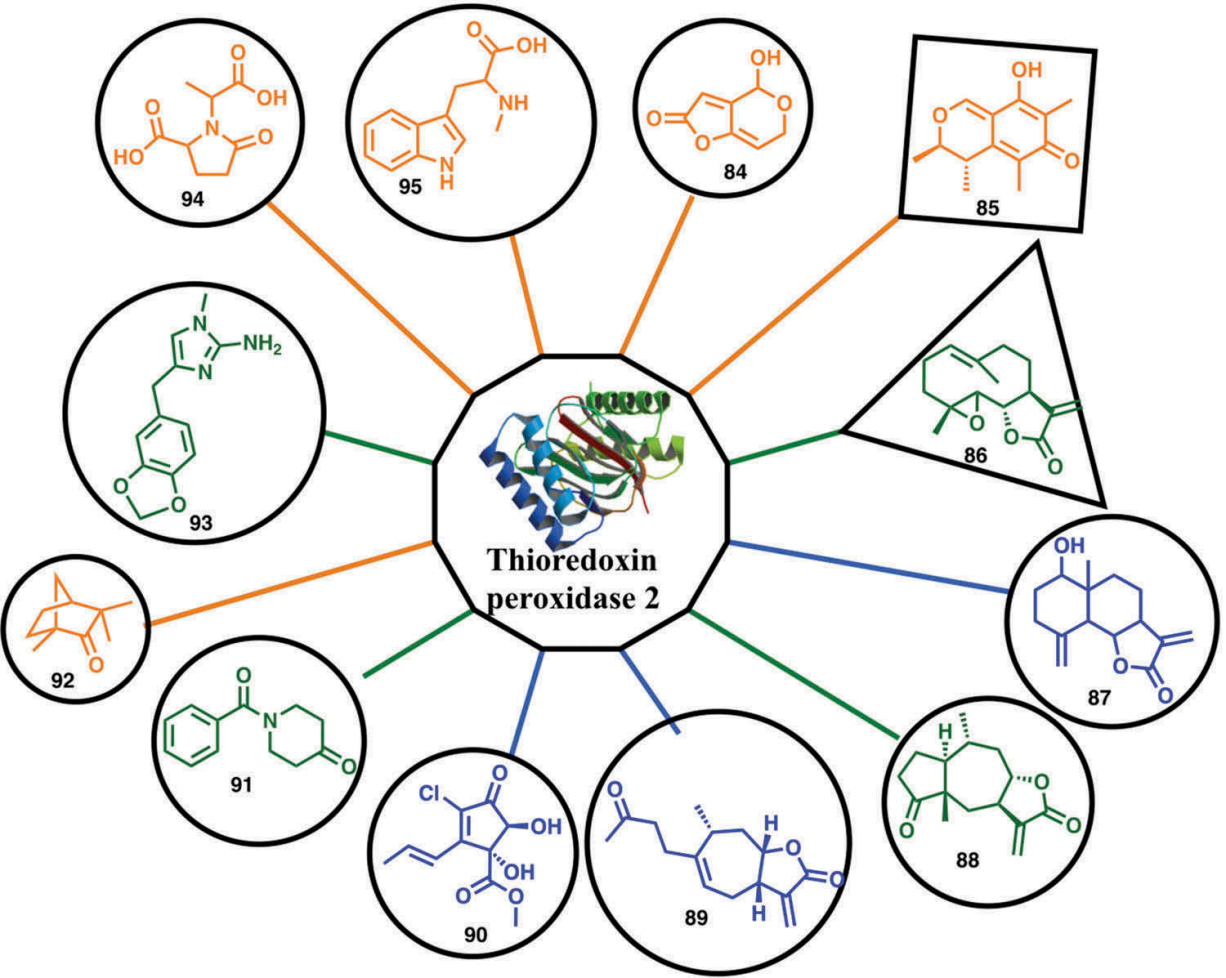
A high ligandable malarial target Thioredoxin peroxidase 2 (Trx-Px2) with its 12 fragment binders (**84**–**95**): green indicates strong binding, blue indicates medium binding and orange indicates weak binding.

The malaria proteome has been examined by a fragment-based approach using native mass spectrometry to detect protein–ligand interactions that exploit proteins produced by structural genomics efforts [30]. Using this assay, a fragment-sized library consisting of 643 natural products with molecular weight <250 were screened against 62 potential protein targets for malaria. Ninety-six (96) low molecular natural products were identified as binding partners of 32 of the putative malarial targets and 79 of these fragments can inhibit the growth of malaria parasites *in vitro* (Figure 15). Fragment hits and proteins were connected to each other based on the binding interaction, resulting in a network with 96 fragments and 32 proteins. There were 48 selective fragments that bound a single protein and 48 fragments that bound more than one protein. The ratios between the protein-ligand complex signal intensity and the unbound protein signal intensity provide a relative ligand affinity ranking, which together with hit rates, defined the protein target ligandability. High ligandable targets (Figure 15) were defined as those with ≥2 strong/medium binders and ≥7 hits (greater than 1% of the library); medium ligandable targets were defined as those with at least 1 strong/medium binder and ≥2 hits; and low ligandable targets were defined as those with at least 1 binder. Nine (9) malarial protein targets with high ligandability were identified and should be considered as prioritized targets in future drug discovery studies.

## 4. Conclusion

**Figure 16.**
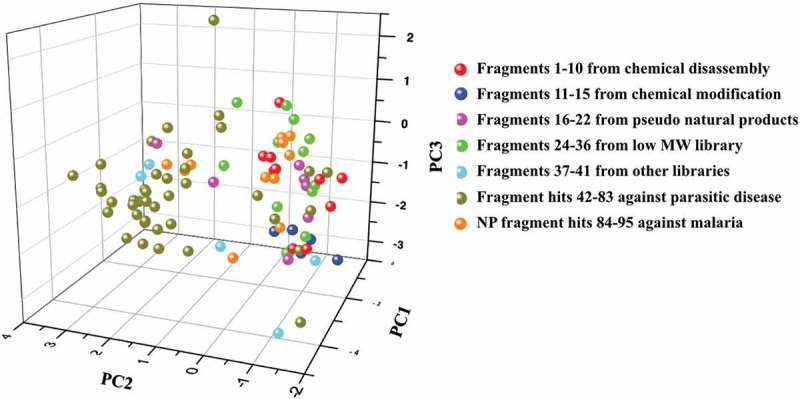
Score plot of ChemGPS-NP analysis of 94 fragments (**1**–**22, 24**–**95**) in this manuscript. PC1 (molecular size) versus PC2 (molecular aromaticity) versus PC3 (molecular lipophilicity) for sets of 10 fragments from chemical disassembly of larger natural products (**1**–**10**) (red), five fragments from chemical modification of small natural products (**11**–**15**) (blue), seven fragments from pseudo natural products (**16**–**22**) (magenta), 13 fragments from a low MW natural product library (**24**–**36**) (green), five fragments from other libraries (**37**–**41**) (cyan), 42 fragment hits against parasitic diseases (**42**–**83**) (dark yellow) and 12 natural product fragment hits against malaria (**84**–**95**) (orange).

Comparison of the fragments highlighted in this opinion piece was analyzed for natural product likeness using Chemical global positioning system-natural product (ChemGPS-NP). It is a widely used chemical positioning system tuned for exploration of biologically relevant chemical space [23]. The ChemGPS-NP space map uses 35 descriptors to evaluate the main rules including aspects of size, shape, lipophilicity, polarity, polarizability, flexibility, rigidity and hydrogen bond capacity [24,53]. Fragments were analyzed and explained by eight respective principal components that could be mapped onto a consistent eight-dimensional map (supplementary table S1). The three most significant PCs explain 71% of the variance and can be interpreted as follows: PC1 represents size, shape, and polarizability, PC2 corresponds to aromatic- and conjugation-related properties, PC3 describes lipophilicity, polarity and H bond capacity [54]. In the resulting plot (Figure 16), although there was an overlap between different datasets, fragments sourced from natural products stretch out in the right side (negative PC2 values), while most of the synthetic fragments are situated in the left region (positive PC2 value). The main loadings for PC2 are aromatic or flat related properties. In other words, natural product fragments versus synthetic fragments contain more three-dimensional structures.

The natural product-based fragment libraries occupied similar areas of chemical space and were differentiated from fragment hits from other fragment libraries that were mainly based on synthetic compounds obeying the Rule of Three. This analysis provided further evidence of the 3D-shape and high Fsp^3^ attributes and confirmed the previous analyzes showing unique chemical diversity.

Protein targets for *T. brucei, T. cruzi*, Dengue virus, and *P. falciparum* have been screened by fragment-based methods. A virtual screen against two Dengue virus targets used a natural product library and a screen against 62 proteins from the malaria proteome used a natural product library.

## 5. Expert opinion

The efforts to develop natural product-based libraries for fragment-based screening have been few and far between. In contrast to the recognition that 3D-space and Fsp^3^ are important in drug leads and candidate drugs, the actual use of the arsenal of compounds produced by nature has been extremely limited. There are many ways to take advantage of the inherent chemical diversity of natural products, such as chemical disassembly, chemical modifications of low MW natural products, synthesis based on scaffolds occurring in natural products, pseudo natural products by combination of natural product scaffolds as well as natural products with low MW.

A major recommendation emerging from this opinion piece is that there are now several well-established methods to take advantage of natural products. Methods based on Scaffold Tree and Scaffold Network analyzes can lead to synthetically accessible natural product-like compounds. The design principles applying to other fragment screening libraries can be applied equally to natural product-based fragment libraries. Retrospective analysis of natural products has shown that a relatively large number of low MW natural products are produced by nature and that separation science could allow even more natural products to be incorporated into fragment screening libraries.

The principle of fragment-based screening allows libraries to contain small numbers of compounds. Rather than limiting library design and the choice of chemical reactions by the need to produce large numbers for HTS library sets, the development of Cluster analysis, as highlighted in this opinion piece, allows selection of diversity in a logical fashion so that libraries can be designed to cover significant chemical diversity.

Efforts need to be devoted to achieving natural product-based library sizes of 2,000–3,000 compounds to allow the full potential of natural products to benefit anti-parasitic drug discovery efforts.

Native mass spectrometry has been used with a natural product fragment library to examine potential drug targets from the malaria proteome. The use of low MW natural products as fragments in native mass spectrometry may represent the largest single dataset disclosed for a fragment-based drug screening campaign with 62 targets [30]. The library contained of only 643 unique low MW natural products [30].

Major efforts to use fragment-based screening for anti-parasitic protein targets should examine significantly more potential drug targets. This approach allows the ligandability of putative targets to be evaluated in a cost-effective fashion. A major recommendation is to screen larger number of potential drug targets in parasitic diseases. Once targets are validated or shown to be druggable in fragment-based screening, they can be explored using HTS campaigns.

At this stage, parasitic disease hit and lead discovery has not utilized the 3D attributes and the high Fsp^3^ characteristics of natural products. We argue that this is a missed opportunity.

Natural products have 3-dimensional shape that may better interact with proteins. Natural products are pre-validated both by the chemical space explored by nature in evolution and an embedded recognition of protein binding sites captured during their biosynthesis. Natural product-like libraries can capture the sp^3^ richness of natural products.

We recommend the use of low MW natural products and low MW natural product-like compounds in fragment-based drug screening for parasitic diseases.

## Article Highlights

- Natural products have 3-dimensional shape that may better interact with proteins.
- Natural products are pre-validated both by the chemical space explored by nature in evolution and an embedded recognition of protein binding sites captured during their biosynthesis.
- Natural product-like libraries can capture the sp^3^ richness of natural products.
- Scaffold tree and scaffold network analysis allows the identification of scaffolds occurring in larger natural products.
- Fragment-based drug discovery has been used as an alternate strategy for drug development for neglected disease.
- The efforts to develop natural product-based libraries have been few and far between, in contrast to the recognition of 3D-space and Fsp^3^ being important in drug leads and candidate drugs.
- The use of natural products and natural product-like libraries has had limited application to parasitic diseases.
- The malaria proteome has been investigated by fragment-based screening using low MW natural products.

This box summarizes key points contained in the article.
